# On-Line Smoothing for an Integrated Navigation System with Low-Cost MEMS Inertial Sensors

**DOI:** 10.3390/s121217372

**Published:** 2012-12-13

**Authors:** Kai-Wei Chiang, Thanh Trung Duong, Jhen-Kai Liao, Ying-Chih Lai, Chin-Chia Chang, Jia-Ming Cai, Shih-Ching Huang

**Affiliations:** 1 Department of Geomatics, National Cheng-Kung University, 1 University Road, Tainan 701, Taiwan; 2 Industrial Technology Research Institute, 195 Chung Hsing Road, Chutung, Hsinchu 310, Taiwan

**Keywords:** on-line smoothing, INS/GPS integration, Kalman filter

## Abstract

The integration of the Inertial Navigation System (INS) and the Global Positioning System (GPS) is widely applied to seamlessly determine the time-variable position and orientation parameters of a system for navigation and mobile mapping applications. For optimal data fusion, the Kalman filter (KF) is often used for real-time applications. Backward smoothing is considered an optimal post-processing procedure. However, in current INS/GPS integration schemes, the KF and smoothing techniques still have some limitations. This article reviews the principles and analyzes the limitations of these estimators. In addition, an on-line smoothing method that overcomes the limitations of previous algorithms is proposed. For verification, an INS/GPS integrated architecture is implemented using a low-cost micro-electro-mechanical systems inertial measurement unit and a single-frequency GPS receiver. GPS signal outages are included in the testing trajectories to evaluate the effectiveness of the proposed method in comparison to conventional schemes.

## 1. Introduction

For navigation applications and the Mobile Mapping System (MMS), the integration of the Inertial Navigation System (INS) using an Inertial Measurement Unit (IMU) and the Global Positioning System (GPS) is widely applied for determining state vectors, which include the position, velocity, and orientation of the mobile platform. The advantages of INS are autonomous operation, high measurement sampling rate, and short-term accuracy. However, its navigation accuracy degrades rapidly with time if no external aiding source is available. This is particularly true when a low-cost IMU is applied. In contrast, GPS is able to provide long-term position and velocity accurately. However, a low sampling rate, environmental dependence, and the lack in orientation determination with single antenna are the primary limitations for navigation oriented-applications with GPS alone. The integration of INS and GPS is an optimal solution that utilizes the advantages of each system and overcome in limitations.

The Kalman filter (KF) [1] is commonly applied for multi-sensor data fusion. The KF aims to find the optimal estimates of the system states based on the minimization of covariance. There are two main steps in the KF computation cycle. In the first step, the prediction primarily relies on the information of the system output. In the second step, whenever aiding measurements are available, the estimates are updated using this information. However, besides the limitations reported in [2–5], most filtering techniques including KF can only be used for optimal estimation when aiding measurements are available. Otherwise, navigation states rely on predicted results from the INS mechanization. This significantly increases positional drifts in the system when a low-cost micro electro mechanical system (MEMS) IMU is applied in GPS-denied environments. In addition, the system and the measurement noise must be carefully pre-modeled for the filtering process. This procedure is costly and impossible to implement in certain cases [2].

To overcome the limitations of filtering techniques, smoothing algorithms have been effectively applied for integrated navigation systems when post-processing is permitted. In principle, smoothing estimates the states at time *k* given the measurements at a time greater than *k*. Most smoothing algorithms utilize forward and backward passes to find the estimates of the states at every epoch of the system output. In the popular Rauch-Tung-Strieble (RTS) smoother [6], the forward estimation is obtained using standard KF and the estimation of the backward pass is based on the maximum likelihood estimates. The main advantages of this algorithm are high reliability and simple implementation. Liu *et al.*[7] developed Two-Filter Smoothing (TFS) and applied it in INS/GPS integration for post-processing applications. The estimation accuracies of TFS and RTS smoother are comparable. The computational times are similar as well. In comparison to forward KF, the improvement of smoothing in positioning error ranges from 35% to 95% depending on the length of GPS signal outages. Chiang [8] proposed a combination of RTS smoothing and artificial neural networks (ANN) for accurate INS/GPS integrated position and orientation determination. The research illustrated that the improvement of ANN-RTS algorithm compared to RTS is about 70%. However, the extra computational time for ANN-RTS algorithm is significant due to the training process.

In general, the estimation accuracy of smoothing is superior to that of filtering. However, most smoothing techniques have been applied for post-processing applications since the backward process always starts from the end of the forward filtering mission. This limits smoothing in real-time applications. The present study utilizes smoothing to on-line update the states of the system for near-real-time applications.

## 2. Optimal Estimations and Problem Statements

### 2.1. Kalman Filter

The KF is considered as a special form of Bayesian estimation [9,10], in which the system and measurement models are originally linear or linearized into linear functions as shown:

(1) $$x_{k}=\Phi_{k-1;k}x_{k-1}+w_{k}$$

(2) $$z_{k}=H_{k}x_{k}+v_{k}$$

where *x_k_* ∈ *R*^*n*_*x*_^ is the state vector at time *k*, Φ*_k_*_−1;_*_k_* is the state transition matrix from epoch *k* − 1 to *k*, *w_k_* ∈ *R*^*n*_*x*_^ is the system noise, *z_k_* ∈ *R*^*n*_*z*_^ is the aiding measurement, *H_k_* is the measurement mapping matrix, and *v_k_* ∈ *R*^*n*_*v*_^ is measurement noise.

In the KF, Gaussian distribution is assumed for the system and measurement noise with zero mean and covariances *Q_k_* and *R_k_*, respectively:

(3) $$w_{k}\sim N\left(0,Q_{k}\right)$$

(4) $$v_{k}\sim N\left(0,R_{k}\right)$$

With this assumption, the prior and posterior probability density function (PDF) of state vector given aiding measurements, *p*(*x_k_* | *z_k_*_−1_), *p*(*x_k_* | *z_k_*) are normal distribution functions.

(5) $$p\left(x_{k}|z_{k-1}\right)=N\left(x_{k};{\hat{x}}_{k|k-1},P_{k|k-1}\right)$$

(6) $$p\left(x_{k}|z_{k}\right)=N\left(x_{k};{\hat{x}}_{k|k},P_{k|k}\right)$$

where *N*(*x_k_*; *x̂_k|k_*, *P_k|k_*) denotes a normal distribution of *x_k_* with mean *x̂_k|k_* and covariance *P_k|k_*.

Derived in terms of the minimum mean square error of the state vector, KF calculation steps are:

Prediction:

(7) $${\hat{x}}_{k|k-1}=\Phi_{k-1;k}{\hat{x}}_{k-1}$$

(8) $$P_{k|k-1}=\Phi_{k-1;k}P_{k-1}\Phi_{k-1;k}^{T}+Q_{k}$$

Updating:

(9) $$K_{k}=P_{k|k-1}H_{k}^{T}{\left[H_{k}P_{k|k-1}H_{k}^{T}+R_{k}\right]}^{-1}$$

(10) $${\hat{x}}_{k}={\hat{x}}_{k|k-1}+K_{k}\left[z_{k}-H_{k}{\hat{x}}_{k|k-1}\right]$$

(11) $$P_{k}=P_{k|k-1}-K_{k}H_{k}P_{k|k-1}$$

where *x̂_k|k_*_−1_, *P_k|k_*_−1_ are the predicted states and covariance at time *k* given information at time *k* − 1, *x̂_k_*_−1_, *P_k_*_−1_ are the estimated states and covariance at time *k −1*, and *x̂_k_*, *P_k_* are the estimated states and covariance at time *k*.

Although well known as an optimal linear estimator, the KF has some limitations for INS/GPS integration. First, KF can only be applied with linear models and the assumption of Gaussian-distribution noise. However, in INS/GPS integration applications, the system and measurement models are originally non-linear and the noise during operation may be non-Gaussian. This is particular true when a low-cost MEMS IMU is used with highly dynamic movement. These restrictions were investigated in [2–5].

Second, in a generic KF, covariance matrices Q and R, which represent the behavior of the system and measurement noise, respectively, must be carefully pre-modeled. Insufficiently known or wrong *a priori* statistics about the system and measurement noise result in poor performance or divergence of the filter [11]. To estimate the *a priori* statistics of noise, intensive calibration or reliable information about the sensors of the system is usually required. This increases the overall cost of the system. This problem is mentioned in [8,11–13].

In addition, since the sampling rate of the INS is higher than that of GPS, the states of the system are updated for the estimates only when GPS measurements are available. Otherwise, the predicted estimates of KF are used. This situation leads to large positional and attitude errors when using a MEMS-based INS/GPS system during long periods of GPS signal outages, as described in [8].

### 2.2. Rauch-Tung-Striebel Off-Line Smoothing

According to [6], the purpose of smoothing is to estimate the PDF of the states at time *k* with all given measurements up to and at time *N*, where *k* ≤ *N*:

(12) $$P\left(x_{k},x_{k+1}|z_{N}\right)=P\left(x_{k+1}|x_{k}\right)P\left(x_{k}|z_{k}\right)P\left(z_{k+1},\ldots ,z_{N}|x_{k+1}\right)P\left(z_{k}\right)$$

The RTS smoother [6] applies the maximum likelihood of the state vectors given aiding measurements vectors as the criteria for finding optimal estimates:

(13) $$\text{max}\;L\left(x_{k},x_{k+1}|z_{N}\right)=\text{max}\;\text{log}\;P\left(x_{k},x_{k+1}|z_{N}\right)$$

where *L*(*x_k_*, *x_k_*_+1_ | *z_N_*) is the likelihood of *x_k_*, *x_k_*_+ 1_ given *z_N_*

By resolving the criteria in Equation (13), the estimates and covariance of the states are obtained:

(14) $${\hat{x}}_{k|N}={\hat{x}}_{k}+C_{k}\left[{\hat{x}}_{k+1|N}-\Phi_{k;k+1}{\hat{x}}_{k}\right]$$

(15) $$P_{k|N}=P_{k}+C_{k}\left[P_{k+1|N}-P_{k+1}\right]C_{k}^{T}$$

where *x̂_k|N_*, *P_k|N_* are the smoothed states and covariance at time *k* given information up to *N* (*k* ≤ *N*), *x̂_k_*, *P_k_* are the states and corresponding covariance estimated by KF at time *k*, *C_k_* is the cross covariance, determined as:

(16) $$C_{k}=P_{k}\Phi_{k;k+1}^{T}P_{k+1}^{-1}$$

**Figure 1.**
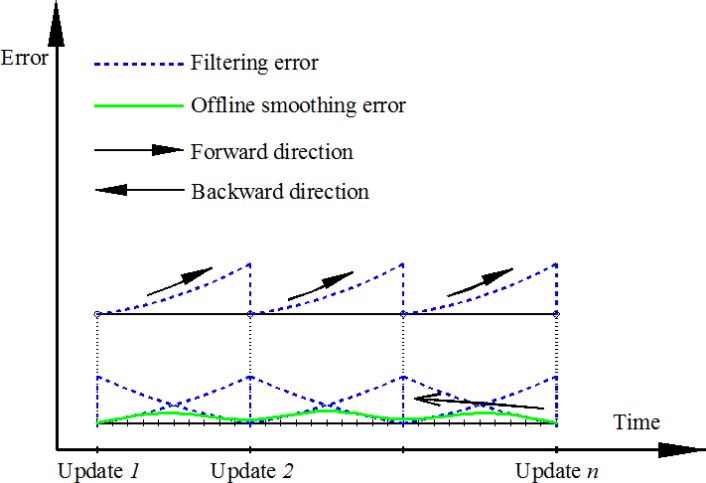
Process and performance of off-line smoothing.

**Figure 2.**
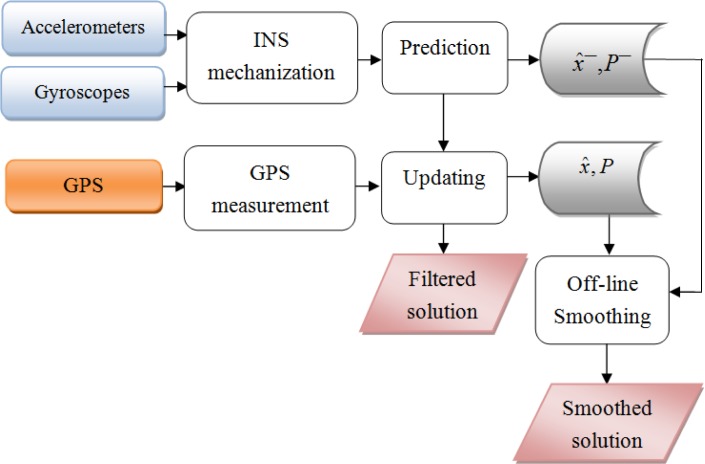
Integrated architecture with KF and off-line smoothing.

The implementation of the RTS smoother includes two main stages of estimation: the forward direction estimation using standard KF and the backward direction using Equations (14) to (16). First, the prediction is implemented based on the output of the system model. The predicted states *x̂*^−^ and covariance *P*^−^ are stored in temporary files. Whenever an aiding measurement is available, the KF is activated. The updated states *x̂* and covariance *P* are calculated accordingly. These parameters are also stored in temporary files for later smoothing. This recursive process continues until the end of the mission, for a forward pass. After the forward pass, the RTS smoothing begins from the end of the mission and moves back to the starting point of the data set. The predicted and updated information are used in this process. This implementation of the RTS smoother has some limitations. First, it is commonly applied for post processing and can be considered as off-line smoothing. Second, it is time-consuming to store and retrieve the predicted and updated information from the stored files. A lot of storage is also required. Third, since the smoothing is activated from the end to the beginning of the mission, the output solution is always in reverse sequence of time. This is inconvenient when the output data is used for other tasks. Figure 1 describes the process and the performance of RTS off-line smoothing and Figure 2 shows the architecture design of INS/GPS integration with off-line smoothing.

## 3. On-Line Smoothing

### 3.1. Principle of On-Line Smoothing

**Figure 3.**
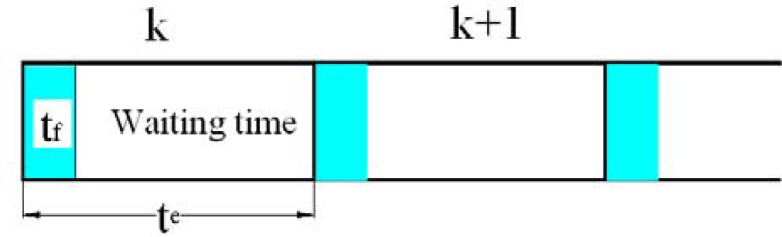
Proportion of time used for filtering in integrated navigation system. t_e_ is the time of each epoch *k*, and t_f_ is time required for filtering.

**Figure 4.**
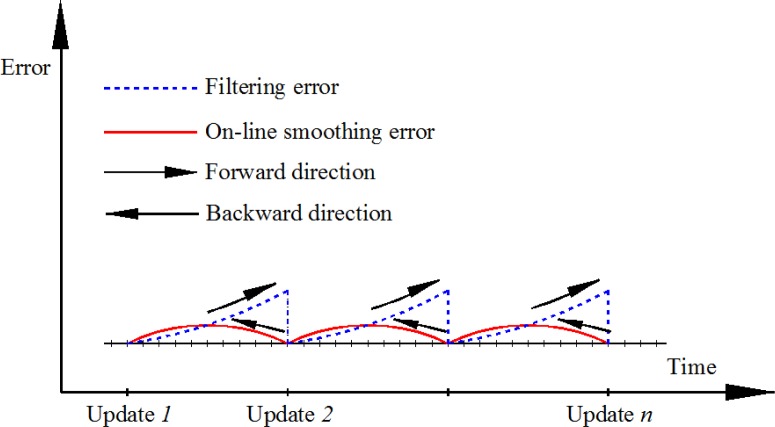
Process and performance of on-line smoothing.

**Figure 5.**
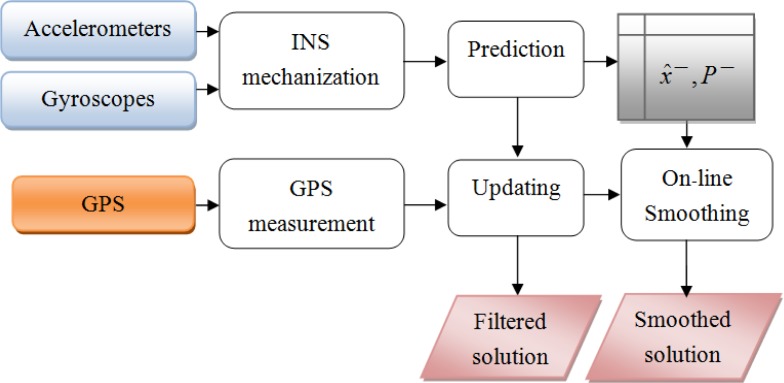
Integrated architecture with KF and on-line smoothing.

The proposed on-line smoothing is implemented during operation time. The algorithm overcomes the disadvantages of off-line smoothing. The proposed on-line smoothing is derived from the RTS smoother algorithm. Instead of waiting until the end of the data set, the smoothing process is activated whenever updating measurements are found. In the KF, the execution time for prediction and filtering is shorter than the time of each epoch (the time between two consecutive IMU samples), meaning that in each epoch, the processing unit waits for the incoming data of the next epoch from an IMU after implementing the KF as shown in Figure 3. The on-line smoothing aims to utilize the time remaining in each epoch to implement smoothing to improve the overall accuracy of the system during operation. Figure 4 illustrates the principle of the proposed on-line smoothing. The architecture design of INS/GPS integration with on-line smoothing is shown in Figure 5.

As shown in Figures 4 and 5, during estimation, the KF is applied for forward estimation; the predicted information is temporally stored in dynamic arrays. When the updated measurement from aiding sensors is available, updating is activated. The RTS smoother performs smoothing from the current time to the previous updating time using the latest updated estimates and predicted information in dynamic arrays. After smoothing process in the current epoch has finished, the smoothed solution is stored or given in output streams with the sequence from the previous updating time. The data in the dynamic arrays is replaced by the predicted information of the next epoch. With this implementation, the smoothing process is nearly parallel to the filtering process.

In real-time applications, the time required for on-line smoothing must be considered to keep track of the continuity of the incoming data from the sensor. The time required for on-line smoothing is closely related to the number of smoothing steps. It thus depends on the sampling rate of the system sensor and the length of time between consecutive measurement updates.

### 3.2. Output Rate of On-Line Smoothing for Real-Time Application

Let *d_s_* be the frequency of the system sensor (INS sensor). The time for each epoch during system operating is:

(17) $$t_{e}=\frac{1}{d_{s}}$$

Let *t_f_* be the time for each filtering step, *T* is the time for each smoothing step, *d_out_* is output data rate (number of output data packages per second) of on-line smoothing, and *n_up_* is number of updating steps during on-line smoothing or window size of on-line smoothing. Time for smoothing, *t_sm_* in each on-line smoothing window will be:

(18) $$t_{sm}=T\cdot d_{\mathrm{out}}\cdot n_{\mathrm{up}}$$

To implement on-line smoothing without missing incoming data from the system sensor of the next epoch, the following criteria must be kept:

(19) $$t_{f}+t_{sm}\leq t_{e}$$

Substituting Equations (17) and (18) into (19) yields:

(20) $$t_{f}+T\cdot d_{\mathrm{out}}\cdot n_{\mathrm{up}}\leq \frac{1}{d_{s}}$$

From Equation (20), the output data rate in on-line smoothing is determined as:

(21) $$d_{\mathrm{out}}\leq \frac{1-d_{s}t_{f}}{Td_{s}n_{\mathrm{up}}}$$

In practice, the time for each estimation step depend on the configuration of the processor, the applied programming language and the optimization of algorithms. In a test with a program written in C++ language, tested on a Core 2 duo 1.86 GHz CPU, the time for each filtering step is 0.3 ms and for each smoothing step is 0.5 ms. If an IMU with 50 Hz of output rate is applied, the output rate of on-line smoothing will be about 40 Hz. In practical, 25 Hz of output rate and window size *n_up_* = 1 is applied for this case.

A discontinuity between estimating epochs may arise since the output rate is not the same as the sampling rare. In this case, the prediction states between discontinuous epochs *k −* 1 and *k + N* cannot be calculated based on Equation (7). They are instead calculated as:

(22) $$x_{k+N}=\Phi_{k-1;k}\Phi_{k;k+1}\ldots \Phi_{k+N-1;k+N}x_{k-1}$$

### 3.3. Output Rate and Window Size of On-Line Smoothing *versus* Navigation Accuracy

**Figure 6.**
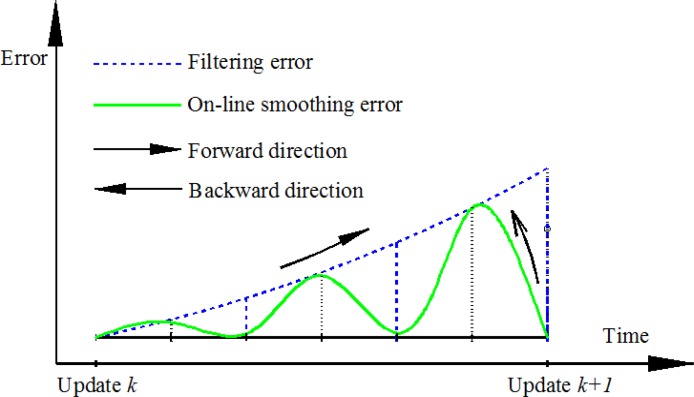
Behavior of on-line smoothing error with discontinuous estimates.

As mentioned in the previous section, to keep track of the incoming data from sensors, the output rate determined by Equation (21) is usually smaller than the sampling rate of the IMU sensor. It means that not all epochs will be estimated by on-line smoothing, therefore the lower the output rate of on-line smoothing is, the worse the overall navigation accuracy becomes. The illustration is shown in Figure 6. However, in some applications, which a high output rate is not required, only smoothed estimates are stored and used as the output solution, thus the good performance of smoothing will be ensured.

In addition, the relationship between navigation accuracy and window size in on-line smoothing algorithm is given in more details. In rationale, the RTS smoother is derived based on the maximum likelihood of the state vectors given aiding measurement vectors as expressed in Equation (13). Now, consider a component in the right side of Equation (12):

It can be seen that *P*(*z_k_*_+1_, ..., *z_N_* | *x_k_*_+1_) = *L*(*x_k_*_+1_ |*z_k_*_+1_, ..., *z_N_*) is the likelihood of state *x_k_*_+ 1_ given aiding measurements *z_i_*(*i* = *k* + 1 → *N*). In estimation manner, the larger range of *i* or the more number of aiding measurements, the better estimates of *x_k_*_+ 1_ or the greater value of likelihood *L*(*x_k_*_+1_ | *z_k_*_+1_, ..., *z_N_*).

**Figure 7.**
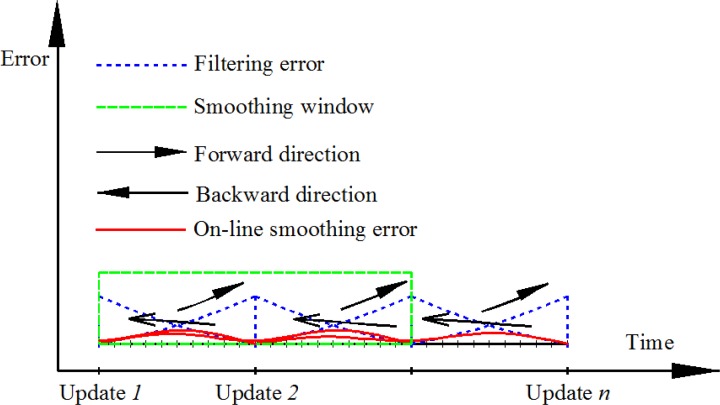
Window size in on-line smoothing.

It means that the greater the smoothing window size applied, the better the obtained estimates become. In the case that the output rate of on-line smoothing is equal to the sampling rate of the system sensor and the smoothing window size is equal to number of updating steps of all data set, it become the off-line RTS smoother. In the on-line smoothing, if the window size greater than 1, overlap parts of two or more smoothing periods are generated, as show in Figure 7. It increases the redundancies for estimation, therefore improving the estimation accuracy.

The component *P*(*x_k_*_+1_ | *x_k_*) = *L*(*x_k_* | *x_k_*_+1_) shown in Equation (12) relates to the relationship between two successively estimated state vectors. Their relationship can be derived based on Equation (7) if they are really successive states. In on-line smoothing not all states may be estimated, the given component become *P*(*x_k_*_+_*_n_* | *x_k_*) = *L*(*x_k_* | *x*_*k*+*n*_) with *n* > 1 and Equation (22) is applied to express their relationship. It is clear that the larger value of *n*, the decreased probability in determination of *x*_*k*+ n_ given *x_k_* or the smaller value of *L*(*x_k_* | *x*_*k*+*n*_), the likelihood of *x_k_* given *x_k_*_+ n_. It means that the lower the smoothing output rate, the worse the estimation accuracy even if only a smoothed solution is considered.

From above analysis, choosing an appropriate parameter is application dependent and can be considered as a critical matter for implementing the on-line smoothing. For applications that require a high output rate, the highest output rate of on-line smoothing must strictly obey Equation (21) and it is updated in each updating step based on given parameters. For applications in which navigation accuracy is preferred rather than a high output rate, the smoothing output rate and window size should be increased to improve the estimation accuracy. On the other hand, output rate and window size are also affected by operating conditions. In the case of long-time GPS signal outages, the window size of on-line smoothing increases naturally. In this case the output rate will be decreased accordingly to keep track the incoming data from sensor. In fact, simultaneously receiving data from sensors, filtering, and on-line smoothing can be implemented by applying parallel computing or multi-thread in programming technique. In this case, the output solution will fulfill both estimation accuracy and high output rate.

## 4. System and Software Design

### 4.1. Equipment

Two INS/GNSS integrated navigation systems were set up to conduct a field test in this research. The reference system comprised a high-end tactical-grade IMU, SPAN-LCI (NovAtel). A dual-frequency geodetic-grade GNSS receiver, ProPak V3 (NovAtel). A distance measurement instrument (DMI). The specifications of SPAN-LCI IMU are shown in Table 1.

**Figure 8.**
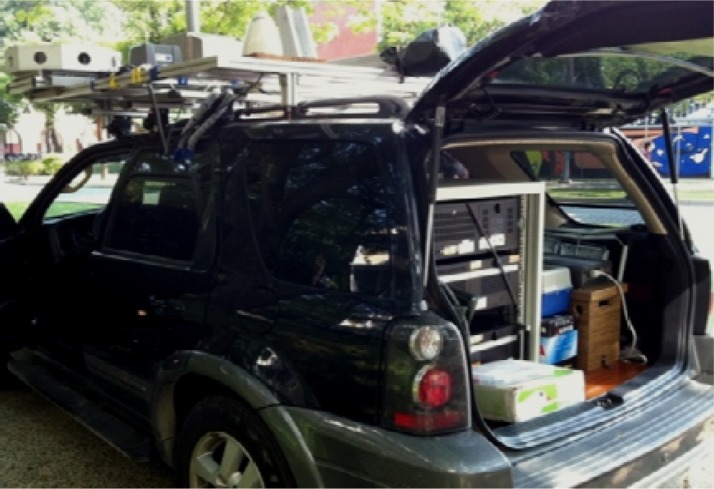
Testing platform.

The testing system comprised a low-cost MEMS IMU, MIDGII (Microbotics), with an integrated single-frequency GPS receiver. The specifications of the testing system are shown in Table 2. Both systems were mounted on a mobile mapping van for data collection to validate the performance of the proposed algorithms, as shown in Figure 8.

### 4.2. Software Design

**Figure 9.**
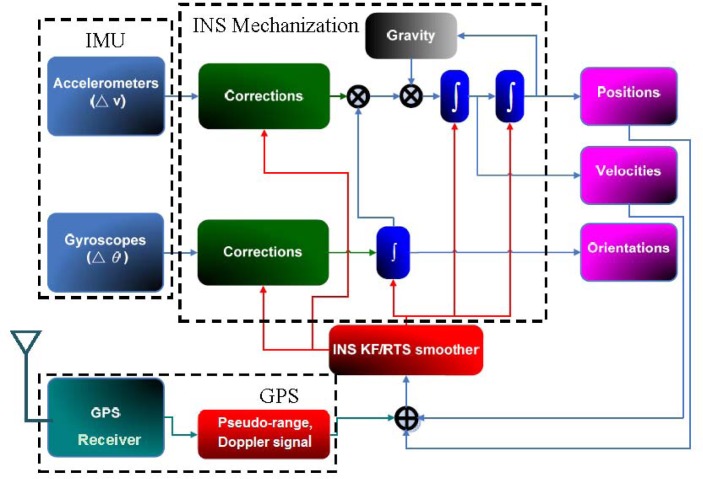
Tightly coupled scheme.

A tightly coupled (TC) INS/GPS integration scheme with KF-based estimation was implemented in this study. As shown in Figure 9, the input data sets for the integrated system were raw IMU and GPS measurements. The raw measurements provided by IMU, including incremental angles and velocities sensed by the gyroscope and accelerometers, after compensation for system errors, are processed by an INS mechanization for a navigation solution in a local-level frame. Pseudo-range and Doppler signal measurements, the raw measurements from GPS, are firstly pre-processed for eliminating system errors such as clock, troposphere, and ionosphere errors. All the measurements are then blended and estimated with the KF and smoothing for the optimal navigation solution, including position, velocity, and orientation. Compared to a loosely coupled (LC) integration scheme, the TC scheme has major advantage of aiding measurements being supported by GPS even when fewer four GPS satellites are available. For a LC scheme, at least four visible GPS satellites are required to derive GPS-aided solutions. Therefore, the TC scheme is particularly suitable for land-based mobile mapping where the system is often operated in GPS-hostile environments such as urban canyons or under dense canopy.

**Figure 10.**
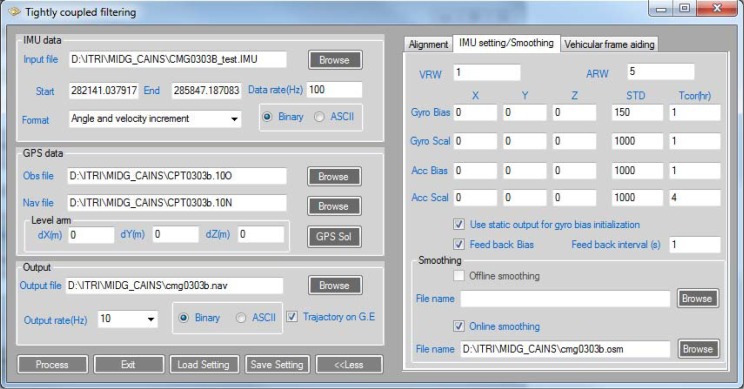
GUI for of tightly coupled integration scheme.

Based on the designated scheme, software for processing raw measurements from GPS and the IMU was developed in the C++ programming language. The graphical user interface (GUI) design is illustrated in Figure 10. The input for the software includes IMU and GPS raw measurements. The software can also process dual-frequency GPS carrier phase measurements in differential mode for accurate GPS solutions.

## 5. Performance Validation of Proposed Algorithms

**Figure 11.**
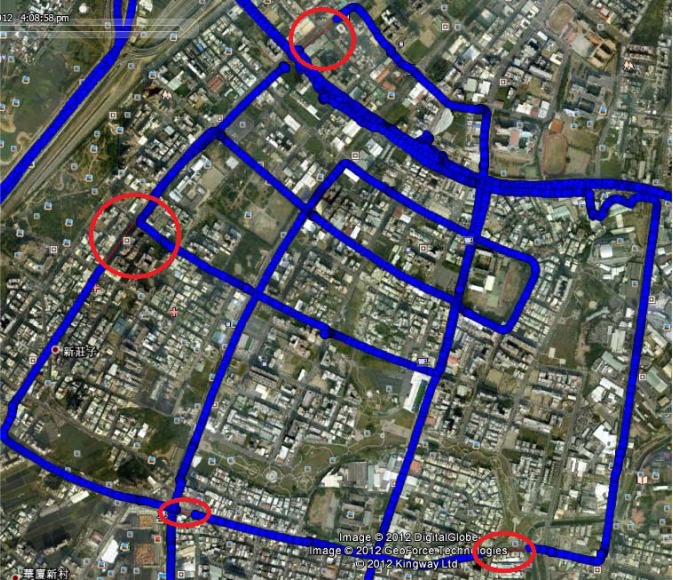
First test trajectory. GPS signal outages are marked by red circles.

In the first test, the testing data sets were collected under various environment scenarios in urban and suburban areas in Kaohsiung, Taiwan. The testing trajectory is shown in Figure 11. The reference trajectory was generated with the reference system with its IMU raw measurements and raw GPS carrier phase measurements processed in differential mode with commercial software, Inertial Explorer (NovAtel), performing sensor fusion in TC smoothing mode with aid from DMI. In general, the kinematic positioning accuracy of the applied reference system was less than 10 centimeters, which is considered sufficient.

For the testing scenario, three algorithms, namely the KF, off-line RTS smoother and, on-line smoothing based on a forward KF and backward RTS, were implemented. The estimated results of the algorithms, including position and orientation, were compared to the reference data for analysis.

**Figure 12.**
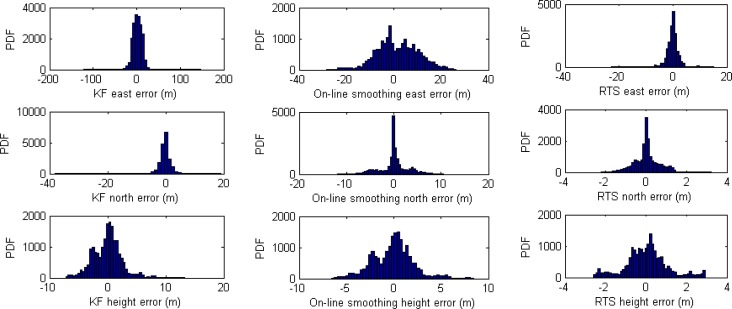
Positional error distributions of KF, on-line smoothing, and RTS smoother.

**Figure 13.**
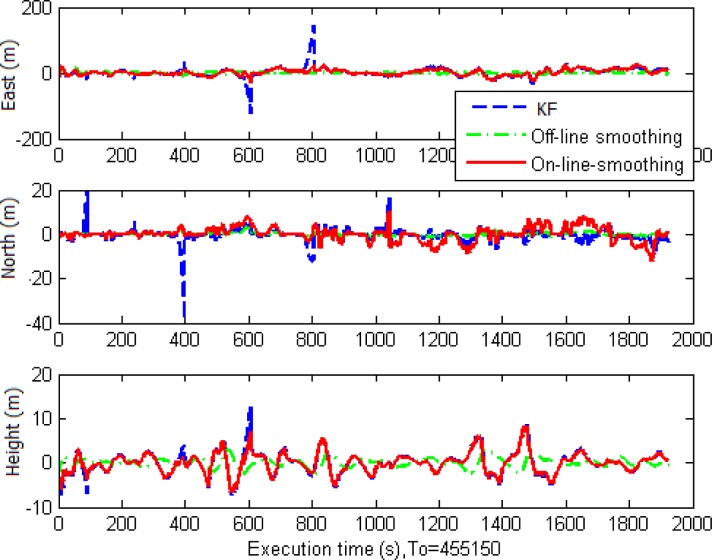
Positional error comparison.

Table 3 and Figure 12 illustrate numerical and histogram statistics of positional root mean square errors (RMSE) and Figure 13 illustrates the performance of the three algorithms. The analysis indicates that in general, the estimation accuracy in terms of position for smoothing is superior to that for KF. The improvement in RMSE is about 85% with RTS smoother and 60% with on-line smoothing. The efficiency can be seen clearly when GPS signals are weak or blocked. Overall, the off-line RTS smoother produced the best estimates. During GPS signal outages, the performances of on-line smoothing and conventional RTS smoother were comparable.

**Figure 14.**
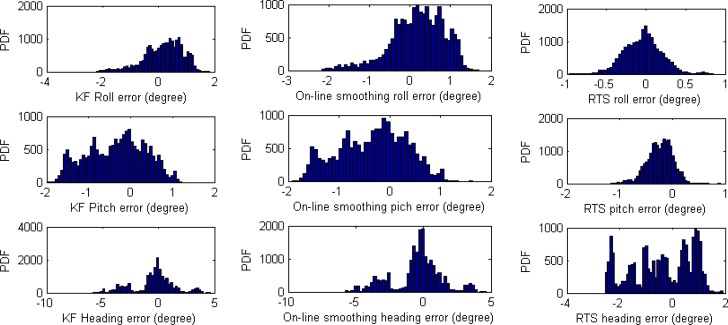
Orientation error distributions of KF, on-line smoothing, and RTS smoother.

**Figure 15.**
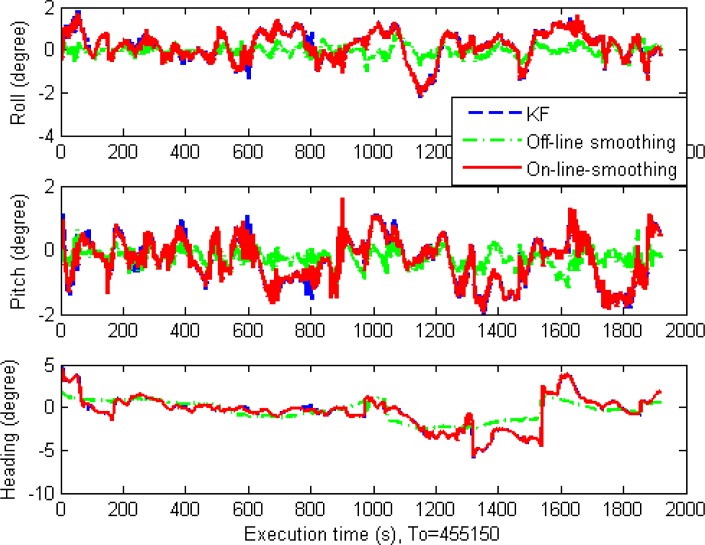
Orientation error comparison.

For orientation accuracy, the improvement of smoothing techniques is not as large as that for positional estimates. The estimated orientation of the off-line RTS smoother is the best, but only slightly. The performances of on-line smoothing and the KF are similar overall. The only difference is that during GPS outages, the estimated results of on-line smoothing are slightly better than those of KF. Table 4, Figures 14 and 15 show these results.

**Figure 16.**
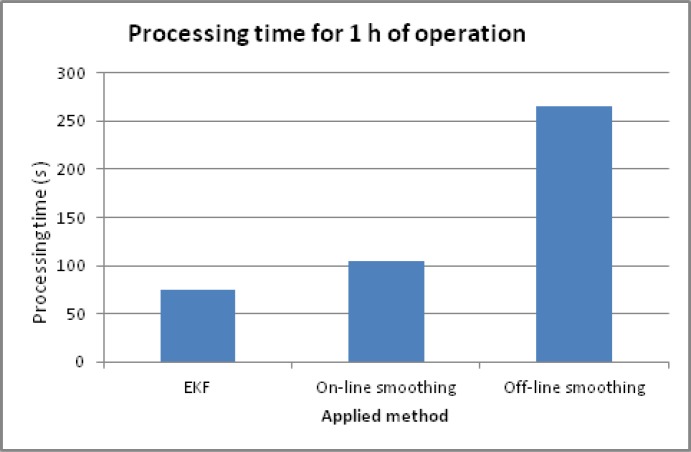
Processing time comparison.

The processing time of the three algorithms was also analyzed. Table 5 shows the numerical analysis. Figure 16 shows a comparison of time for KF, off-line RTS smoother, and on-line smoothing. The statistic data is surveyed from the processing time for a data set of 1 h on-field operation time, a 50-Hz IMU sampling rate, and processed on a computer with a Core 2 duo 1.86 GHz CPU. The analysis illustrates that although off-line smoothing produced the best estimates, it required the longest processing time. The time increment of off-line RTS smoothing is about 250% compared to that of KF. The time increment of on-line smoothing compared to that for KF is only about 40%, for an improvement in positional accuracy of about 60%. In addition, the estimated output of on-line smoothing is near real-time and the data is in a forward sequence of time, which is convenient for data usage.

**Figure 17.**
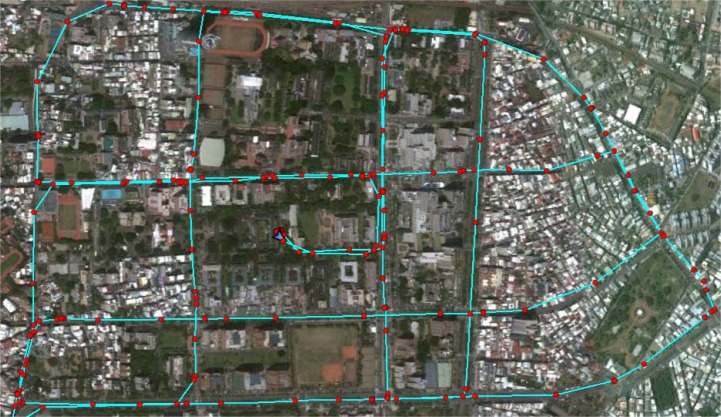
The second test trajectory, red points are GPS updates.

**Figure 18.**
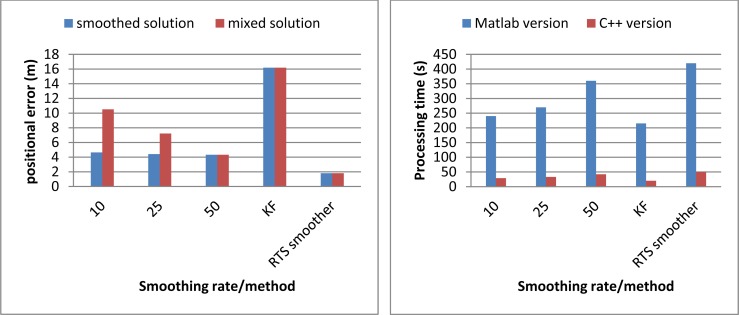
Relationship between smoothing rate and positional error and processing time.

**Figure 19.**
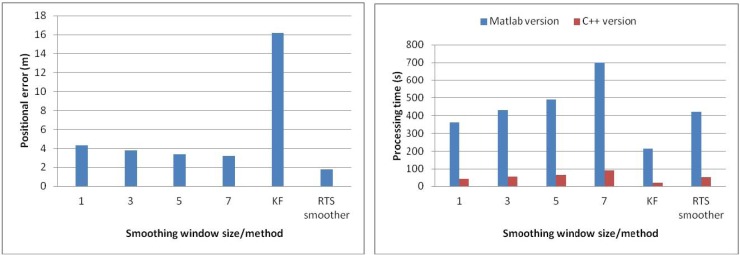
Relationship between smoothing window size and positional error and processing time.

The second test was conducted to analyze the relationship between output rates, window size and estimation accuracy in on-line smoothing. The data was collected with the same systems applied in the previous test in Tainan, Taiwan. The testing trajectory is shown on Figure 17. Updating rate from GPS is reduced to 0.05 Hz (20 s for each GPS update). Three cases of online-smoothing output rate with 50 Hz, 25 Hz and 10 Hz are tested, two types of output solutions including smoothed only solutions and mixed solutions (mixes of smoothed and filtered solutions) are considered. The processing time tracked on two versions of software is tested, C++ and Matlab, the operation time for testing was 34 min. Table 6 and Figure 18 show the testing results. Four options of on-line smoothing window size with 50 Hz output are also considered and illustrated in Table 7 and Figure 19.

The second test results indicate that if the smoothed solutions are considered as the output solutions, there are only small variations in estimation accuracy with different smoothing output rates. On the other hand, the accuracy of mixed solutions decreases dramatically if output rates decrease. The processing time changes proportionally with the change of smoothing output rates. On the other hand, the estimation accuracy improves when the smoothing window size increases. However, long processing time causes the latency in the case of large window size. Table 7 and Figure 19 show that the improvement in terms of positioning accuracy with different window size is not significant while the processing time increases dramatically. Tables 6 and 7 also illustrate that by improving the algorithms and programming environment, the processing time decreases significantly.

## 6. Conclusions

This study developed an on-line smoothing algorithm to improve the estimation accuracy of positional and orientation parameters of integrated navigation systems utilizing low-cost MEMS inertial sensor in near-real-time.

The results indicate that the proposed on-line smoothing outperforms the KF. In terms of positional RMSE, the improvement of on-line smoothing is about 60% compared to KF. The performance of on-line smoothing is comparable to that of off-line smoothing.

The estimation accuracy and processing time in on-line smoothing depend on the output data rate and window size accordingly. The optimal parameters for on-line smoothing are application dependent. For the future works, parallel computing technique should be investigated and applied on on-line smoothing to improve both output rate and estimation accuracy.

## Figures and Tables

**Table 1.** Reference system specifications.

**Physical characteristics**	**IMU performance**		**Measurement accuracy**	

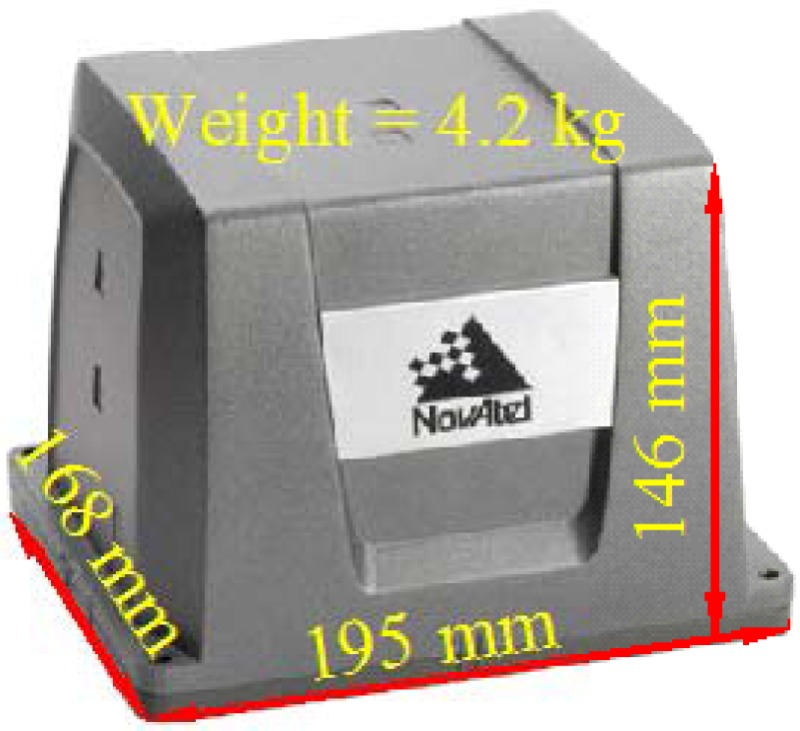	Output rate (Hz)	200	Heading (degree)	0.02
	Gyro bias (degree/h)	1.0	Attitude (degree)	0.4
	Gyro scale factor (ppm)	100	Position (m)	0.1–1.5
	Accelerometer bias (mg)	1.0	Velocity (m/s)	0.02
	Accelerometer scale factor (ppm)	250	Altitude (m)	0.08

**Table 2.** Testing system specifications.

**Physical characteristics**	**IMU performance**		**Measurement accuracy**	

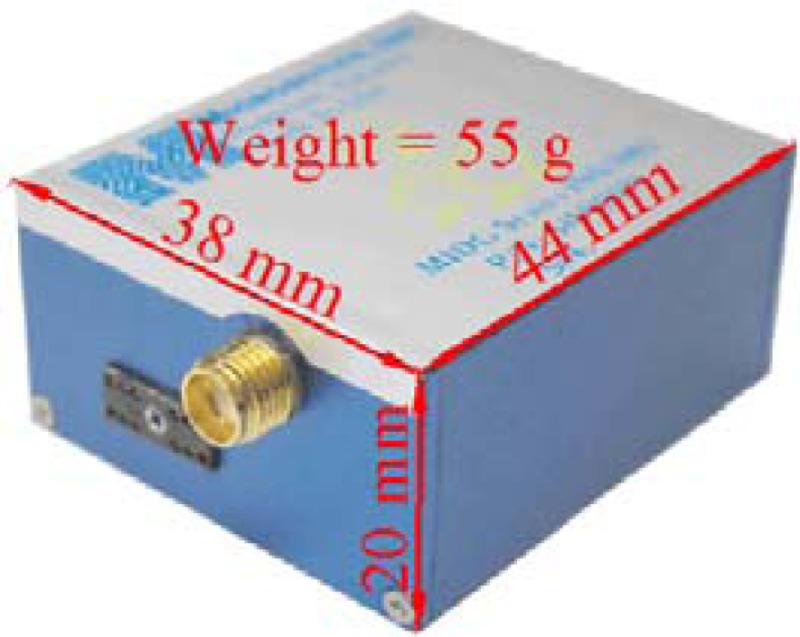	Output rate (Hz)	50	Heading (degree)	2
	Gyro bias (degree/h)	4.7	Attitude (degree)	0.4
	Gyro scale factor (ppm)	5,000	Position (m)	2–3
	Accelerometer bias (mg)	6.0	Velocity (m/s)	0.2
	Accelerometer scale factor (ppm)	19,700	Altitude (m)	3–5

**Table 3.** Comparison of three estimation strategies in terms of positional error.

**Method**	**RMSE (m)**				**Improvement (%)**
	**East**	**North**	**Up**	**3D**	

KF	17.423	3.437	2.462	17.93	
Off-line smoothing	2.449	0.688	0.946	2.71	85
On-line smoothing	7.196	2.511	2.063	7.9	56

**Table 4.** Comparison of three estimation strategies in terms of orientation error.

**Method**	**RMSE (degree)**		
	**Roll**	**Pitch**	**Heading**

KF	0.71	0.77	1.90
Off-line smoothing	0.25	0.34	1.21
On-line smoothing	0.68	0.73	1.82

**Table 5.** Comparison of processing time.

**Method**	**Processing time (s)**	**Increment (%)**
KF	75	
On-line smoothing	105	40
Off-line smoothing	265	253

**Table 6.** Relationship between smoothing rate and positional error and processing time.

**Smoothing rate (Hz)**	**Positional error (m)**		**Processing time (s)**	
	**Smoothed solution**	**Mixed solution**	**Matlab version**	**C++ version**

10	4.64	10.51	240	29
25	4.42	7.22	270	33
50	4.33	4.33	360	42
KF	16.19	16.19	215	20
RTS smoother	1.81	1.81	420	50

**Table 7.** Relationship between smoothing window size and positional error and processing time.

**Smoothing window size**	**Positional error (m)**	**Processing time (s)**	
		**Matlab version**	**C++ version**

1	4.33	360	42
3	3.80	430	52.5
5	3.35	490	63.7
7	3.17	700	91
KF	16.19	215	20
RTS smoother	1.81	420	50
